# Focal pulsed field ablation for guiding and assessing the acute effect of cardioneuroablation

**DOI:** 10.1007/s10840-023-01716-4

**Published:** 2024-01-29

**Authors:** Ivan Sikiric, Zrinka Jurisic, Toni Breskovic, Marina Juric-Paic, Nina Berovic, Josip Kedzo, Ivan Pletikosic, Tolga Aksu, Ante Anic

**Affiliations:** 1 Department of Cardiology, University Hospital Centre Split, Split, Croatia; 2 Department of Cardiology, Yeditepe University Hospital, Istanbul, Turkey

As a relatively new treatment option, cardioneuroablation (CNA) aims to modify the behavior of the cardiac autonomic nervous system to prevent some/all of the autonomic processes occurring in vasovagal syncope (VVS) and functional bradyarrhythmias by using endocardial ablation techniques [1–5]. The fundamental approach of CNA involves pinpointing clusters of autonomic ganglia called ganglionated plexi (GPs), situated within epicardial atrial fat pads. There are three main approaches for identification of GPs: high-frequency stimulation (HFS)-guided, anatomy-guided, and spectral mapping/fractionation-guided approaches [1–5]. Focal pulsed field ablation (PFA) has emerged as a cutting-edge technology in the field of cardiac electrophysiology, offering a promising alternative to the thermal ablative modalities for the treatment of cardiac arrhythmias [6]. Here, we propose another application of this exciting tool, a novel approach aimed at guiding and evaluating the immediate effect of CNA.

Two patients in their early 40 s were referred to our center after experiencing multiple episodes of cardio inhibitory VVS (CI-VVS) over the past 3 months, confirmed through head-up tilt-test for one patient and implantable loop recorder (ILR) for the other. The third patient, a 35-year-old female, had been admitted for the third time in the last 6 months due to symptomatic sinus bradycardia, accompanied by significant premature ventricular contractions (PVCs) that exacerbated her symptoms. Following thorough discussions about various management options, CNA with PFA guidance was proposed as a therapeutic intervention. Here, we present our proposed workflow.

Under conscious sedation and local anesthesia, access was obtained via a right femoral venous approach. High-resolution electroanatomic mapping (PentaRay Nav, Biosense Webster, Irvine, CA, USA) of the left atrium was performed using a three-dimensional mapping system (Carto 3, Biosense Webster, Irvine, CA, USA). Subsequently, an irrigated ablation catheter (Thermocool ST nonSF, D-type, Biosense Webster, Irvine, CA, USA) was introduced into the left atrium and connected to the CENTAURI PFA generator (Galvanize Therapeutics, San Carlos, CA, USA). Initially, focal PFA (25 A/30 pulses) was applied to the anterosuperior aspect of the right superior pulmonary vein ostium, assumed to be an anatomical marking of the superior right atrial GP (RSGP), to provoke transient vagal response (VR) and bradycardia (Fig. 1A). Following this, radiofrequency ablation (RFA) with a targeted ablation index of up to 550 was applied in the described segment with the same catheter, progressing ostial and antral towards the interatrial septum. During RFA, the sinus rate increased, and PVCs were suppressed/not detectable after the first lesion. In a single patient, a similar amplitude of sinus bradycardia was induced after initial RFA lesion set at the same anatomical site using focal PFA. Subsequently, we proceed with RFA targeting the right side, aligning with our original plan for biatrial ablation. The ablation catheter was then introduced into the right atrium, in an area that is anatomically nearly inseparable from the left set of lesions, resulting in a significant increase in sinus rate. The right set of lesions was consolidated to a total of 6 lesions. Afterward, no sinus bradycardia was provoked with focal PFA on the right side and finally on the left side at the same position as at the beginning (Fig. 1C and D). This represented an acute endpoint and suggested ablation of the RSGP. During the observation period, sinus rhythm was maintained, and no ventricular ectopic activity was detected. For the post-procedural follow-up assessment, the patient exhibiting bradycardia and PVCs underwent a 24-h Holter ECG monitoring 2 months after the initial intervention. The analysis revealed a normal heart rate, without ventricular ectopy. For the remaining two patients, one underwent a repeat HUTT one week after the procedure, while the other continued his clinical follow-up with regular IRL examinations. Notably, the result of the HUTT was negative, indicating the absence of cardio-inhibitory response (Table 1).

**Fig. 1:**
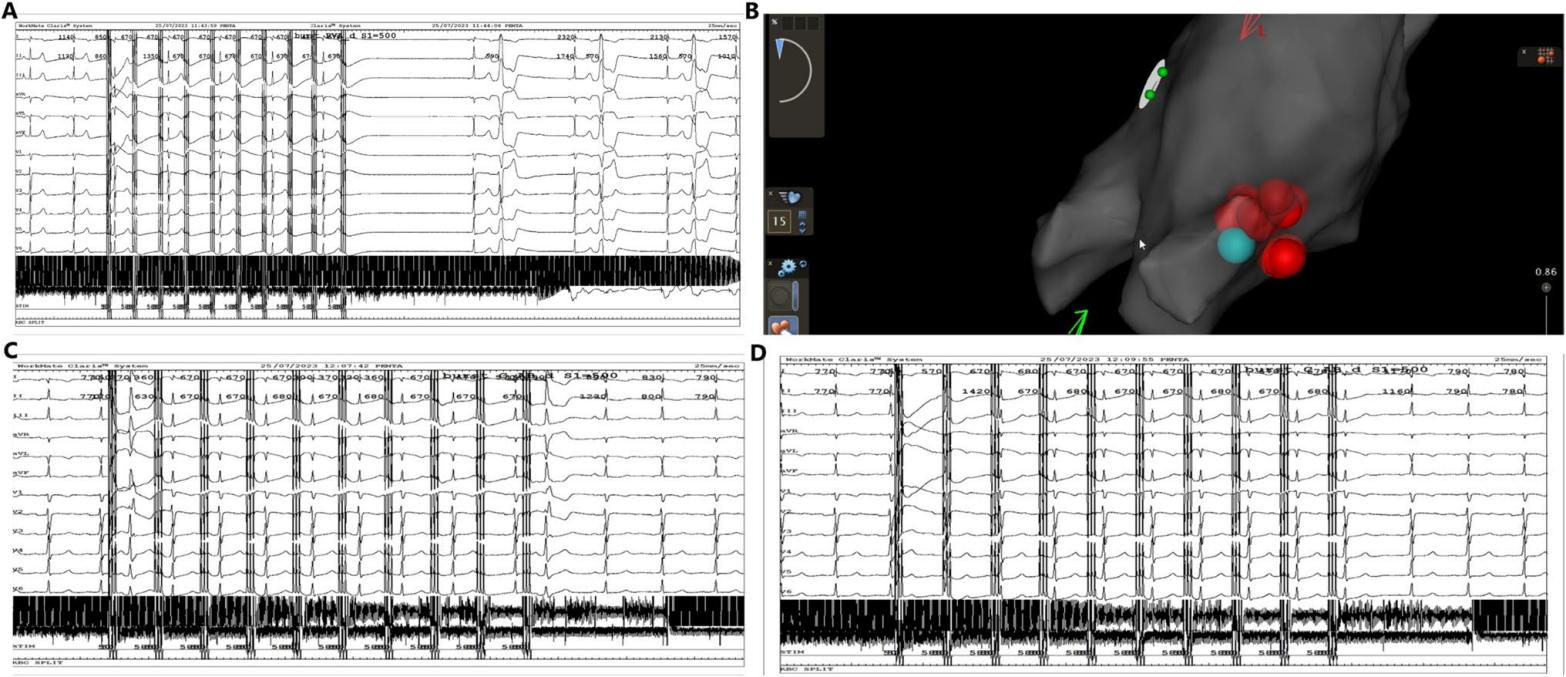
**A** First PFA application from left atrium inducing vagal response. **B** Full lesion set on 3D EAM from superior view. **C** PFA from right atrium after last RFA. **D** PFA from left atrium after last RFA

**Table 1:** Patient characteristics and follow-up details

Patient (n)	1	2	3
Gender	Female	Female	Female
Age	42	40	35
Diagnosis method	ILR	HUTT	24-h Holter ECG
Follow-up duration*	1 month	2 months	3 months
Follow-up method	ILR monthly examination	HUTT—1 week after procedure	24-h Holter ECG—2 months after procedure

*at the time of drafting this research letter

The PFA could achieve pulmonary vein isolation (PVI) without any of collateral damage in patients with paroxysmal AF, including vein stenosis, phrenic nerve injury, or esophageal damage [6]. However, authors demonstrated vagal responses (VR) during PFA energy delivery. Whether the VRs are nerve damage or a neurological stress response due to electrical stimulation like HFS response was unclear. Recently, Reddy et al. [7] assessed the impact of PFA on the GPs in patients undergoing PVI. In the prospective phase, pre-PFA HFS identified 65 GP sites. During PFA, vagal effects were noted in 45% of first PF applications, persisting through all applications in 83%. HFS post-PFA reproduced vagal effects in 29 of 38 sites (76%) in low-voltage tissue. Unlike with thermal ablation, between baseline and 3 months, heart rate did not increase in PFA arm.

Our initial experience demonstrates the potential of focal PFA guided CNA as an effective therapeutic strategy for patients suffering from functional bradyarrhythmia and CI-VVS. Through meticulous patient selection and the integration of cutting-edge technologies such as PFA into the CNA procedure, we have successfully guided and assessed the acute effects of this intervention. The outcomes in our patients, particularly the resolution of symptomatic sinus bradycardia and suppression of PVCs, highlight the efficacy of this combined approach. Also, there might be another positive aspect of this approach, as focal PFA could enhance RFA penetration to epicardial tissues. This remains an intriguing area for further investigation.

While our experience provides valuable insights into the immediate effects of PFA-guided CNA, small sample size, involving only three patients, necessitates cautious interpretation of the results. Long-term follow-up studies and larger patient cohorts are essential to establish the durability and safety of this approach.

## Supplementary Information

Below is the link to the electronic supplementary material.

Video 1 Transient vagal response observed during the mapping of RSGP, following focal PFA (MP4 26170 KB)

## Data Availability

The data presented in this research letter are derived from the clinical experiences and interventions performed at our center, involving three patients undergoing cardioneuroablation (CNA) guided by focal pulsed field ablation (PFA). The electroanatomic mapping data, ablation procedures, and follow-up assessments were conducted using commercially available equipment, including the PentaRay Nav and Carto 3 mapping systems, the Thermocool ST nonSF ablation catheter, and the CENTAURI PFA generator. However, due to the sensitive and personal nature of patient health data, the full dataset, including detailed patient records, electroanatomic maps, and specific procedural parameters, cannot be made publicly available. Patient privacy and confidentiality are of utmost importance, and ethical considerations prohibit the release of individual-level data. Researchers interested in collaborating, verifying, or replicating aspects of this study are encouraged to contact the corresponding author for potential collaboration and data-sharing arrangements. Access to de-identified summary data, aggregated results, or specific aspects of the methodology may be considered on a case-by-case basis, subject to approval from the institutional review board and compliance with relevant ethical and legal standards. We are committed to promoting transparency and reproducibility in research, and we welcome inquiries regarding the study design, methodology, and analysis procedures. The corresponding author will provide reasonable assistance and information to facilitate further scientific inquiry while respecting the ethical principles governing patient confidentiality and privacy.

## References

[1] Pachon JC, Pachon EI, Cunha Pachon MZ, Lobo TJ, Pachon JC, Santillana TG. Catheter ablation of severe neurally meditated reflex (neurocardiogenic or vasovagal) syncope: cardioneuroablation long-term results. Europace. 2011;13:1231–42. 21712276 10.1093/europace/eur163

[2] Aksu T, Padmanabhan D, Shenthar J, Yalin K, Gautam S, Valappil SP, Banavalikar B, Guler TE, Bozyel S, Tanboga IH, Lakkireddy D, Olshansky RB, Gopinathannair R. The benefit of cardioneuroablation to reduce syncope recurrence in vasovagal syncope patients: a case-control study. J Interv Card Electrophysiol. 2022;63(1):77–86. 33527216 10.1007/s10840-020-00938-0

[3] Aksu T, Gopinathannair R, Bozyel S, Yalin K, Gupta D. Cardioneuroablation for treatment of atrioventricular block. Circ Arrhythm Electrophysiol. 2021;14(9): e010018. 34465122 10.1161/CIRCEP.121.010018

[4] Hu F, Zheng L, Liang E, Ding L, Wu L, Chen G, Fan X, Yao Y. Right anterior ganglionated plexus: the primary target of cardioneuroablation? Heart Rhythm. 2019;16(10):1545–51. 31330187 10.1016/j.hrthm.2019.07.018

[5] Piotrowski R, Baran J, Sikorska A, Krynski T, Kulakowski P. Cardioneuroablation for reflex syncope: efficacy and effects on autonomic cardiac regulation-a prospective randomized trial. JACC Clin Electrophysiol. 2023;9(1):85–95. 36114133 10.1016/j.jacep.2022.08.011

[6] Reddy VY, Dukkipati SR, Neuzil P, Anic A, Petru J, Funasako M, Cochet H, Minami K, Breskovic T, Sikiric I, Sediva L, Chovanec M, Koruth J, Jais P. Pulsed field ablation of paroxysmal atrial fibrillation: 1-year outcomes of IMPULSE, PEFCAT, and PEFCAT II. JACC Clin Electrophysiol. 2021;7(5):614–27. 33933412 10.1016/j.jacep.2021.02.014

[7] Musikantow DR, Neuzil P, Petru J, Koruth JS, Kralovec S, Miller MA, Funasako M, Chovanec M, Turagam MK, Whang W, Sediva L, Dukkipati SR, Reddy VY. Pulsed field ablation to treat atrial fibrillation: autonomic nervous system effects. JACC Clin Electrophysiol. 2023;9(4):481–93. 36752473 10.1016/j.jacep.2022.10.028

